# An Atypical Presentation of Bartter Syndrome Type 3 With Hypocalciuria and Opisthotonus Posture in a Preterm Infant

**DOI:** 10.1002/ccr3.70725

**Published:** 2025-07-31

**Authors:** Karim Hassan, Imad Afara, Ali Harajli, Jawad Allam, Kaity Saliba, Frederic Harb

**Affiliations:** ^1^ Faculty of Medicine and Medical Sciences University of Balamand, Main Campus Koura North Governorate Lebanon; ^2^ Gilbert and Rose‐Marie Chaghoury School of Medicine Lebanese American University, Byblos Campus Beirut Beirut Governorate Lebanon

**Keywords:** Bartter Syndrome Type 3, genetic mutation CLCNKB, hypocalciuria, neonatal electrolyte imbalance, opisthotonus posture, pediatric

## Abstract

This case highlights an unusual presentation of Bartter Syndrome Type 3 marked by hypocalciuria and opisthotonus posture. Recognizing such atypical neuromuscular signs is essential for early diagnosis, genetic confirmation, and targeted management in pediatric patients with electrolyte imbalances.

## 1 Introduction

Bartter Syndrome, first designated and named after the American Endocrinologist Frederic Bartter in 1962 [1], is a rare group of autosomal recessive genetic disorders that disrupt chloride absorption in the thick ascending limb of the loop of Henle, with an estimated prevalence of 1/1,000,000 individuals [1]. The condition arises from a defect in the sodium–potassium–chloride cotransporter in this segment of the nephron, and results in excessive urinary loss of chloride, sodium, and potassium, as well as impaired sodium reabsorption. This leads to natriuresis and volume depletion, which in turn triggers the activation of the renin‐angiotensin‐aldosterone system (RAAS) [2]. The subsequent renal vasoconstriction and elevated aldosterone levels, alongside the increased potassium and hydrogen excretion, contribute to causing hypokalemia and metabolic alkalosis [2]. Additionally, this defective function of the cotransporter causes a reduction in paracellular reabsorption of calcium, leading to a cascade of hypercalciuria, hypocalcemia, nephrocalcinosis, and kidney stones [3]. A systematic review conducted by Qasba et al. (2023) reported hypokalemia as the most frequent clinical finding (77.9%), followed by metabolic alkalosis (66.9%), hyperaldosteronism (59.3%), hyperreninemia (58.5%), and hyponatremia (54.2%). Less commonly, presentations included hypochloremia, observed in 50% of the cases, while hypercalciuria and hypomagnesemia were reported in only 12.2% and 9.3% of the cases, respectively [4].

Clinical features of Bartter Syndrome can manifest as early as the antenatal period, often manifesting as polyhydramnios and preterm birth. Additional symptoms include severe polyuria and polydipsia, muscle atrophy and weakness, failure to thrive and developmental delay, dysmorphic facial features and symptoms of renal colic [5]. Atypical features include low urinary sodium and potassium excretion, and absence of metabolic alkalosis, chronic pancreatitis, and neurometabolic syndrome presentation [5]. Several types and subtypes of Bartter Syndrome were classified based on their molecular pathogenesis [6]. Cunha and Heilberg have compiled all types and subtypes of Bartter Syndrome that are autosomal recessive with the exception of Transient BS due to X‐linked mutation in “MAGE‐D2” gene and Autosomal Dominant hypocalcemic hypercalciuria caused by “CASR” gene mutation, based on their genes, inheritance patterns, proteins, and clinical features [6] (Rf. Table 1).

**TABLE 1:** Types of Bartter Syndrome and clinical manifestations.

	Type I	Type II	Type III	Type IVa	Type IVb	Transient BS	AD hypocalcemic hypercalciuria
Gene	*SLC12A1*	*KCNJ1*	*CLCNKB*	*BSND*	*CLCNKA CLCNKB*	*MAGE‐D2*	*CASR*
Inheritance	AR	AR	AR	AR	AR	XLR	AD
Protein	NKCC2	ROMK1	CLC‐Kb	Barttin	CLC‐Ka CLC‐Kb	MAGE‐D2	CaSR
Clinical features	Prematurity Polyhydramnios Nephrocalcinosis Hypokalemic alkalosis Hyposthenuria	Prematurity Polyhydramnios Nephrocalcinosis Hypokalemic alkalosis Hyposthenuria transient Hyperkalemia	Hypokalemia Hypochloremic alkalosis	Prematurity Polyhydramnios Sensorial deafness Hypokalemia Hypochloremic alkalosis	Prematurity Polyhydramnios Sensorial deafness Hypokalemia Hypochloremic alkalosis	Transient salt wasting Polyhydramnios	Hypocalcemic Hypercalciuria

Abbreviations: AD, autosomal dominant; AR, autosomal recessive; XLR, X‐linked recessive.

The diagnosis of Bartter Syndrome can be done prenatally and in the postnatal period. In cases where there is a family history of Bartter Syndrome or high‐risk pregnancy due to early‐onset polyhydramnios, the diagnosis of Bartter Syndrome can be established by assessing aldosterone levels, which usually are increased, in amniotic fluid and fetal cord blood [7]. In the postnatal period, suspected cases of Bartter Syndrome are typically evaluated using routine laboratory tests, which include measuring renal function, serum electrolytes (including magnesium), acid base status, serum renin and aldosterone levels, fractional excretion of chloride (typically > 1%), urinary calcium excretion, and renal ultrasound [8]. Uric acid levels are also considered as key diagnostic markers as hyperuricemia was reported in up to 50% in cases of Bartter Syndrome by Meyer et al. [9].

It is important to mention that a key difference between Bartter Syndrome and Gitelman Syndrome is that patients with Bartter Syndrome have an increased fractional clearance of chloride upon administration of thiazide diuretics as opposed to patients with Gitelman Syndrome which experience a blunted response [10]. However, hallmark and confirmatory diagnostic test remains genetic analysis, whether performed prenatally or in the postnatal period [11]. Despite this, approximately 25% of Bartter Syndrome cases are clinically diagnosed without a genetic testing [12]. Whole Exome Sequencing (WES) is valuable in confirming the subtype of Bartter Syndrome and ruling out mimicking diseases such as Gitelman Syndrome [10]. As for treatment and management, the mainstay of therapy includes oral potassium chloride supplementation alongside potassium‐sparing diuretics and prostaglandin inhibitors such as indomethacin [13].

In the following section, we present and explore a rare and distinct clinical case of Bartter Syndrome Type 3 with an unusual neuromuscular manifestation.

## 2 Case Presentation/Examination

### 2.1 Clinical Features

We present a case of a 2‐month‐old male infant, born preterm at 35 weeks of gestation to a healthy mother with a normal course of pregnancy via uncomplicated vaginal delivery. The birth weight was 2300 g, and the infant was breastfed exclusively since birth. The patient had no known allergies to any food or drug and had an incomplete vaccination record. Due to the normal course of pregnancy and the absence of a family history of genetic diseases, no amniotic fluid study was done.

The patient presented to the emergency department with a 1‐day history of fever, which was unresponsive to antipyretics, reaching 39.4°C, and a 3‐day history of decreased oral intake along with four episodes of normally appearing vomiting and watery, non‐bloody, non‐mucoid diarrhea. Upon physical examination, the patient appeared lethargic, with pale skin, decreased skin turgor, and opisthotonus posture with spastic muscle movement. The rest of the physical examination was normal. Vital signs showed a normal heart rate of 140 beats per minute, a normal temperature of 37.8°C, normal diastolic blood pressure of 42 mmHg, but low systolic blood pressure of 64 mmHg (normal range: 72–104 mmHg). Oxygen saturation was decreased at 88% on room air (normal range: 95%–100% on room air). The infant's weight at admission was 1815 g, indicating a weight loss of 485 g since birth. Based on this clinical presentation, the patient was admitted to the Neonatal Intensive Care Unit for evaluation and management.

## 3 Methods

### 3.1 Investigation and Treatment

Comprehensive laboratory tests were ordered, including Complete Blood Count (CBC), Serum Chemistry and Electrolytes, Urine Chemistry, Liver Function Tests, Creatinine, Blood Urea Nitrogen (BUN), Cholesterol and Triglyceride Panel, and Blood and Urine Cultures.

The results of the laboratory tests included several out‐of‐range results, noting a low red blood cell count, low hemoglobin and hematocrit, along with thrombocytosis and neutrophilia. As well, the patient presented a hypokalemia, hyponatremia, and hypochloremia, along with metabolic alkalosis and hypocalciuria. Moreover, hypercalcemia and hypomagnesemia were shown. The urine chemistry analysis revealed elevated phosphate and chloride levels, with negative blood and urine cultures for both bacterial and parasitic infections.

To address the severe anemia, the patient was transfused with 20 cc/kg of irradiated, anticoagulated, and plasmolyzed packed red blood cells. During the stay in the Neonatal Intensive Care Unit, the patient was put on an ad libitum diet and was supplemented with NaCl, KCl, and MgSO_4_ in maintenance fluids to correct electrolyte and nutrient abnormalities, along with oral SiderAL Forte for iron and vitamin C supplementation. A summary of laboratory findings, including the reference ranges, is provided in the following Table 2.

**TABLE 2:** Laboratory test results and weight.

Parameter	At admission	At discharge	Normal range
Weight	1815 g	2150 g	
RBC	2.32 × 10^6^/mm^3^	3.63 × 10^6^/mm^3^	2.7–4.5 × 10^6^/mm^3^
WBC	9.89 × 10^3^/mm^3^	10.32 × 10^3^/mm^3^	5.0–15.0 × 10^3^/mm^3^
HGB	6.5 g/dL	8.9 g/dL	9.0–14.1 g/dL
HCT	19.4%	28.2%	28%–41%
PLT	537 × 10^3^/μL	626 × 10^3^/μL	150–450 × 10^3^/μL
Neutrophils	77%	48.1%	40%–60%
Lymphocytes	20.2%	45.8%	20%–50%
Monocytes	2.5%	5.9%	2%–10%
Eosinophils	0%	0%	0%–5%
Basophils	0.2%	0.2%	0%–1%
ANC	7.02 × 10^3^/μL	4.96 × 10^3^/μL	1.5–8.0 × 10^3^/μL
ALC	2 × 10^3^/μL	4.73 × 10^3^/μL	1.5–4.5 × 10^3^/μL
BUN	13 mg/dL		7–20 mg/dL
Creatinine	0.3 mg/dL	0.2 mg/dL	0.2–0.58 mg/dL
Na^+^	119 mEq/L	124 mEq/L	130–140 mEq/L
K^+^	2.9 mEq/L	4 mEq/L	3.5–5.0 mEq/L
Cl^−^	60 mEq/L	81 mEq/L	90–110 mEq/L
CO_2_	47 mEq/L	35 mEq/L	35–45 mEq/L
Ca^2+^	11.3 mg/dL	9.4 mg/dL	8.8–10.6 mg/dL
Mg^2+^	1.3 mg/dL	0.7 mg/dL	1.6–2.3 mg/dL
CRP	2 mg/dL		< 10 mg/dL
Urine chemistry			Urine chemistry
Na^+^	79 mEq/L		50–130 mEq/L
Cl^−^	106 mEq/L		95–105 mEq/L
K^+^	75.6 mEq/L		20–100 mEq/L
Ca^2+^	48.4 mEq/L		100–300 mEq/L
${\mathrm{PO}}_{4}^{3-}$	277 mg/dL		4.0–7.0 mg/dL
Uric acid	351 mg/dL		250–750 mg/dL

Abbreviations: ALC, absolute lymphocyte count; ANC, absolute neutrophil count; BUN, blood urea nitrogen; CRP, C‐reactive protein; HCT, hematocrit; HGB, hemoglobin; PLT, platelets; RBC, red blood cells; WBC, white blood cells.

**FIGURE 1:**
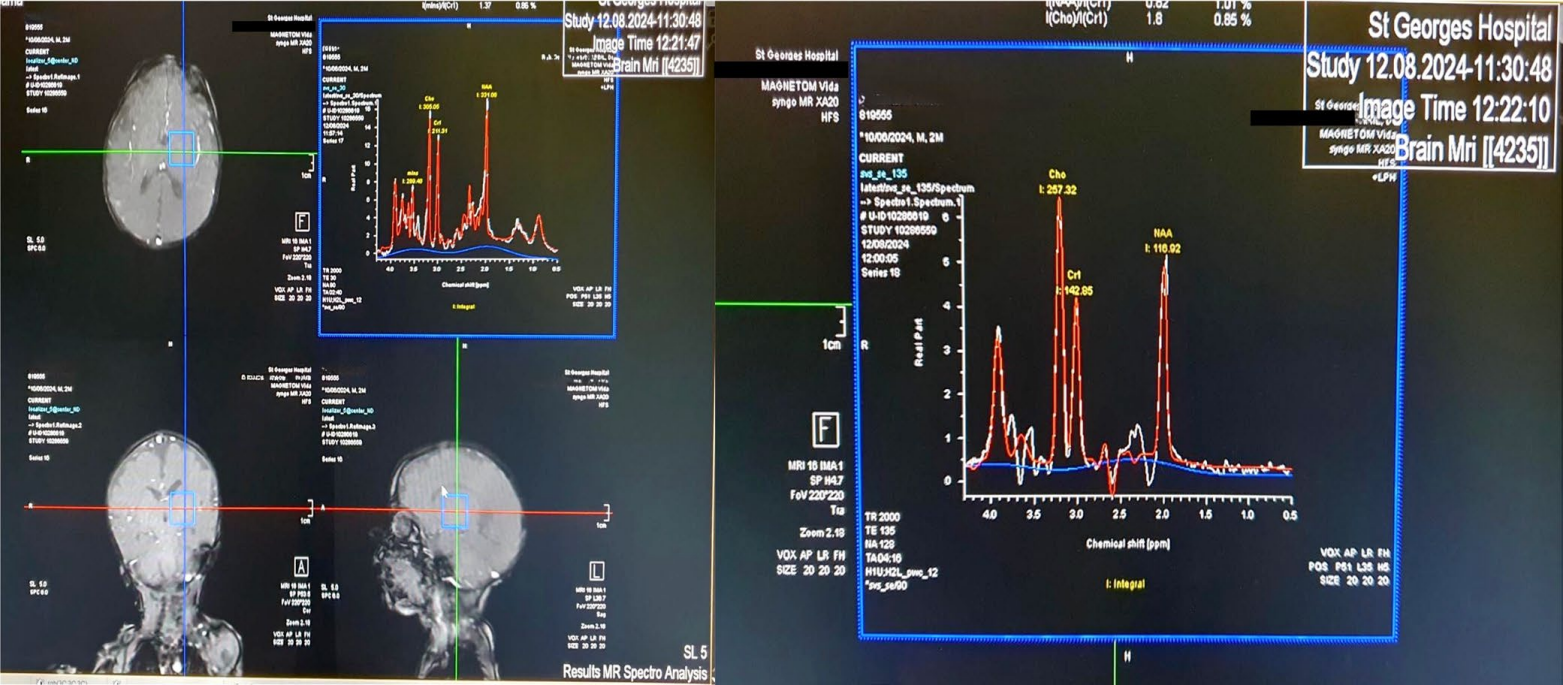
MRI spectroscopy—normal brain metabolic pattern: MRI spectroscopy showed no metabolic abnormalities, supporting the hypothesis that neurological symptoms were metabolic in origin rather than structural. The right panel (A) shows representative MR spectra with metabolic peaks corresponding to Choline (Cho), Creatine (Cr), *N*‐Acetylaspartate (NAA), and other metabolites. The left panel (B) demonstrates the voxel positioning (blue square) in axial, sagittal, and coronal planes, indicating the precise location from which metabolic data were acquired. Peak integrals are provided, indicating relative concentrations within the sampled voxel. Parameters used in this analysis were repetition time (TR) of 2000 ms, echo time (TE) of 135 ms, voxel size of 20 × 20 × 20 mm, and number of acquisitions (NA) of 128. The spectra illustrate typical metabolic profiles, aiding in the identification of potential abnormalities such as altered metabolite ratios that might suggest pathology.

On the fourth day of hospitalization, a chest X‐ray was performed to ensure that no underlying infectious, respiratory, or cardiac complications are contributing or complicating the electrolyte imbalance. Additionally, an electroencephalogram and an MRI Spectroscopy were also conducted to provide metabolic insights into the neurological symptoms such as opisthotonus and lethargy, to rule out any metabolic encephalopathy or brain injury, and to support the conclusion that the neurological symptoms are secondary to the severe electrolyte imbalance. The MRI Spectroscopy (Figure 1) showed normal brain metabolic pattern without evidence of any abnormality. Similarly, the chest X‐ray and EEG, showed no abnormalities, which strengthened the primary diagnosis of Bartter Syndrome.

### 3.2 Differential Diagnosis

Given the patient's clinical presentation of hyponatremic, hypokalemic, hypochloremic metabolic alkalosis, Bartter Syndrome was considered as a potential diagnosis. Although the newborn presented with opisthotonus and spastic muscular activity, which are typically associated with tetanus, this diagnosis was ruled out for several reasons. There was no history of an entering wound or umbilical infection, and the lack of classic symptoms such as trismus, risus sardonicus, or generalized muscle spasms further reduced the likelihood of tetanus. Additionally, there were no signs of autonomic instability, such as respiratory failure. Instead, the neurological symptoms, including lethargy and opisthotonus, were attributed to severe electrolyte imbalances specifically hypokalemia, hyponatremia, and hypomagnesemia linked to an underlying diagnosis of Bartter Syndrome. This metabolic cause was confirmed by normal EEG and MRI spectroscopy results, which ruled out structural or viral encephalopathy. Meningitis was not considered initially since the clinical signs such as low‐grade fever, irritability, and seizures were absent, and inflammatory markers like CRP were low with negative blood and urine cultures. The resolution of the neurological symptoms after correcting the electrolyte imbalances, coupled with the lack of infection indicators, led the medical team to confidently attribute the symptoms to a metabolic disturbance, making meningitis or tetanus infection an unlikely diagnosis.

To confirm the diagnosis, the most logical next step was to perform a “Whole Exome Sequencing” (WES) genetic test to analyze pathogenic variants involved in autosomal recessive, autosomal dominant, and X‐linked diseases. The results revealed that the patient was homozygous for a variant in the “CLCNKB” gene. According to the predictive algorithms for variant impact on protein structure and function, as well as the recommendations of the “American College of Medical Genetics” (ACMG) [14], the variant was classified as Likely Pathogenic (Rf. Table 3).

**TABLE 3:** Whole exome sequencing data.

Gene	cHGVS	pHGVS	Zygosity	Classification
*CLCNKB*	NM_000085.5:c.97C>T	p.Arg33*	Homozygous	Likely pathogenic

Abbreviations: cHGVS, coding Human Genome Variation Society; pHGVS, protein Human Genome Variation Society.

## 4 Conclusion and Results

This case presents with hallmark features of type III Bartter Syndrome, including hypokalemic, hypochloremic, and hyponatremic metabolic alkalosis, along with hypomagnesemia. What makes this case particularly noteworthy is the presence of hypocalciuria and opisthotonus—a clinical manifestation not previously reported in association with this syndrome. In our patient, opisthotonus, characterized by a pronounced arching of the back, serves as a striking indicator of underlying neuromuscular dysfunction linked to profound electrolyte disturbances. This rigid posturing, commonly seen in severe neurological conditions, aligns here with classic Bartter‐related abnormalities such as hypokalemia and metabolic alkalosis. Although opisthotonus may appear in other clinical contexts, its occurrence alongside muscular spasms, elevated urinary phosphate, and potassium depletion strongly reinforces the diagnosis of Bartter Syndrome. This observation underscores the neuromuscular consequences of severe metabolic imbalance and highlights the importance of recognizing atypical presentations in rare genetic disorders to inform both diagnosis and management. Moreover, this patient's case highlights the importance of genetic testing in confirming the diagnosis of Bartter Syndrome, particularly with the existence of challenges posed by atypical presentations that overlap with other genetically inherited diseases like Gitelman Syndrome. The opisthotonus feature presented in this patient is what makes this case a unique and rare case of Bartter Syndrome. Once faced with such rare and phenotypically overlapping cases, comprehensive history‐taking and extensive physical examination are key to facilitating diagnosing and managing such rare disorders.

Therefore, early recognition and intervention can have an important role in mitigating complications and improving patients' outcomes. Hence, the WES remains the gold standard tool for diagnosis, ensuring precise genetic classification and facilitating appropriate clinical management.

## 5 Discussion

Bartter Syndrome of Type III is an autosomal recessive disorder caused by mutations in the “CLCNKB,” which encodes the chloride channel protein “CLC‐Kb,” predominantly expressed in the kidneys [4]. This channel plays a pivotal role in the reabsorption of NaCl, which is essential for the urine concentration and the maintenance of systemic blood pressure [4]. Disruption of “CLC‐Kb” function can lead to salt‐wasting nephropathy, manifesting as renal salt loss, hypokalemic metabolic alkalosis, and hypercalciuria, key characteristics of Bartter Syndrome type III [4]. The genomic variant “c.97C>T” is a point mutation localized in the coding sequence of the “CLCNKB” gene. This single nucleotide substitution results in the change of a codon from “CGA” to “TGA” at the amino acid position 33, generating a “Premature Termination Codon” (PTC), denoted as “p. Arg33*” [12]. The presence of a PTC is predicted to results in either the production of a truncated protein or the activation of nonsense‐mediated mRNA decay (NMD), a cellular mechanism that degrades mRNAs containing PTCs to prevent the synthesis of potentially deleterious truncated proteins [4, 12].

Our case displays classical symptoms of type III Bartter Syndrome including hypokalemic, hypochloremic, and hyponatremic metabolic alkalosis, as well as hypomagnesemia. However, it is distinctive due to the presence of hypocalciuria and opisthotonus posture, which have not been yet described in the literature. In this case, opisthotonus is a valuable and distinguishing symptom associated with electrolyte imbalances in Bartter Syndrome. The infant's rigid arching posture indicates significant neuromuscular dysfunction, which typically comes accompanied by hypokalemia and metabolic alkalosis—the syndrome's hallmarks. While opisthotonus can occur in other disorders, its presence here, coupled with muscular spasms, increased urine phosphate, and hypokalemia, clearly suggests Bartter Syndrome. This clinical result highlights the neuromuscular implications of severe electrolyte imbalance in this rare genetic illness, emphasizing its relevance in guiding diagnosis and care. Regarding calciuria, a cohort study by Castaño et al., examining 30 patients with different mutations in the “CLCNKB” gene, detected hypocalciuria in 23% of the cases, whereas 31% exhibited hypercalciuria [15]. Similarly, a study conducted by Seys et al., analyzing 115 patients with “CLCNKB” mutations, found that 26% of patients with a Gitelman‐like phenotype presented with hypocalciuria [16].

A review of the literature reveals several cases of Bartter Syndrome reported with neurological and neuromuscular symptoms, but none describing opisthotonus posture, which highlights the uniqueness of this case. Anirvan et al. described a case of a 4‐month‐old male infant with electrolyte imbalances characteristic of Bartter Syndrome, presenting with a protruding tongue, suggestive of a neurometabolic disorder [5]. Another case report described a 45‐year‐old woman with sensorineural blindness, muscular weakness, and cramps, later diagnosed with adult‐onset Bartter Syndrome, and found to have mitochondrial myopathy upon muscle biopsy [17]. Verma et al. reported a case of a 10‐year‐old short‐statured boy with polyuria and polydipsia followed by generalized tonic–clonic seizures and quadriparesis diagnosed with Bartter Syndrome. Neurological symptoms were attributed to cytotoxic edema as a result of metabolic derangement and neuronal cellular edema [18]. A case report by Patra et al. described a case of a 4‐month‐old male with only status epilepticus on presentation and was found to have Bartter Syndrome [19].

## Author Contributions

**Karim Hassan:** conceptualization, investigation, visualization, writing – original draft, writing – review and editing. **Imad Afara:** investigation, visualization, writing – original draft, writing – review and editing. **Ali Harajli:** investigation, visualization, writing – original draft, writing – review and editing. **Jawad Allam:** investigation, visualization, writing – original draft, writing – review and editing. **Kaity Saliba:** investigation, visualization, writing – original draft, writing – review and editing. **Frederic Harb:** supervision, visualization, writing – original draft, writing – review and editing.

## Consent

Written informed consent was obtained from the patient's parents in accordance with the journal's patient consent policy. A translated version of the original consent form (in Arabic) has been provided for review purposes.

## Conflicts of Interest

The authors declare no conflicts of interest.

## Data Availability

All data generated or analyzed during this study are included in this published article. No additional datasets were generated or used.

## References

[1] B. H. Lee , H. Y. Cho , H. Lee , et al., “Genetic Basis of Bartter Syndrome in Korea,” Nephrology, Dialysis, Transplantation: Official Publication of the European Dialysis and Transplant Association—European Renal Association 27, no. 4 (2012): 1516–1521, 10.1093/ndt/gfr475. 21865213

[2] G. Deschênes and M. Fila , “Primary Molecular Disorders and Secondary Biological Adaptations in Bartter Syndrome,” International Journal of Nephrology 2011, no. 396 (2011): 209, 10.4061/2011/396209. PMC3177086 21941653

[3] H. W. Seyberth and K. P. Schlingmann , “Bartter‐ and Gitelman‐Like Syndromes: Salt‐Losing Tubulopathies With Loop or DCT Defects,” Pediatric Nephrology (Berlin, Germany) 26, no. 10 (2011): 1789–1802, 10.1007/s00467-011-1871-4. 21503667 PMC3163795

[4] R. K. Qasba , A. C. F. Bucharles , M. V. F. Piccoli , et al., “Bartter Syndrome: A Systematic Review of Case Reports and Case Series,” Medicina (Kaunas, Lithuania) 59, no. 9 (2023): 1638, 10.3390/medicina59091638. 37763757 PMC10537044

[5] P. Anirvan , P. Bharali , M. Gogoi , D. Meher , K. R. Dash , and S. P. Singh , “Chronic Pancreatitis With Type V Bartter Syndrome: An Unusual Presentation,” Clinics and Research in Hepatology and Gastroenterology 46, no. 3 (2022): 101849, 10.1016/j.clinre.2021.101849. 34920143

[6] T. D. S. Cunha and I. P. Heilberg , “Bartter Syndrome: Causes, Diagnosis, and Treatment,” International Journal of Nephrology and Renovascular Disease 11 (2018): 291–301, 10.2147/IJNRD.S155397. 30519073 PMC6233707

[7] H. Shalev , M. Ohaly , I. Meizner , and R. Carmi , “Prenatal Diagnosis of Bartter Syndrome,” Prenatal Diagnosis 14, no. 10 (1994): 996–998, 10.1002/pd.1970141017. 7899275

[8] O. Boyer , F. Schaefer , D. Haffner , et al., “Publisher Correction: Management of Congenital Nephrotic Syndrome: Consensus Recommendations of the ERKNet‐ESPN Working Group,” Nature Reviews Nephrology 17, no. 6 (2021): 434, 10.1038/s41581-021-00431-5. PMC8329697 33941901

[9] W. J. Meyer, 3rd , J. R. Gill, Jr. , and F. C. Bartter , “Gout as a Complication of Bartter's Syndrome. A Possible Role for Alkalosis in the Decreased Clearance of Uric Acid,” Annals of Internal Medicine 83, no. 1 (1975): 56–59, 10.7326/0003-4819-83-1-56. 1147437

[10] K. Nozu , K. Iijima , K. Kanda , et al., “The Pharmacological Characteristics of Molecular‐Based Inherited Salt‐Losing Tubulopathies,” Journal of Clinical Endocrinology and Metabolism 95, no. 12 (2010): E511–E518, 10.1210/jc.2010-0392. 20810575

[11] J. H. Stein , “The Pathogenetic Spectrum of Bartter's Syndrome,” Kidney International 28, no. 1 (1985): 85–93, 10.1038/ki.1985.123. 4046329

[12] E. J. Ashton , A. Legrand , V. Benoit , et al., “Simultaneous Sequencing of 37 Genes Identified Causative Mutations in the Majority of Children With Renal Tubulopathies,” Kidney International 93, no. 4 (2018): 961–967, 10.1016/j.kint.2017.10.016. 29398133

[13] R. Verberckmoes , B. B. van Damme , J. Clement , A. Amery , and P. Michielsen , “Bartter's Syndrome With Hyperplasia of Renomedullary Cells: Successful Treatment With Indomethacin,” Kidney International 9, no. 3 (1976): 302–307, 10.1038/ki.1976.33. 940272

[14] S. Richards , N. Aziz , S. Bale , et al., “Standards and Guidelines for the Interpretation of Sequence Variants: A Joint Consensus Recommendation of the American College of Medical Genetics and Genomics and the Association for Molecular Pathology,” Genetics in Medicine 17, no. 5 (2015): 405–424, 10.1038/gim.2015.30. 25741868 PMC4544753

[15] A. G. Castaño , G. P. de Nanclares , L. Madariaga , et al., “Poor Phenotype–Genotype Association in a Large Series of Patients With Type III Bartter Syndrome,” PLoS One 12, no. 3 (2017): e0173581, 10.1371/journal.pone.0173581. 28288174 PMC5348002

[16] E. Seys , O. Andrini , M. Keck , et al., “Clinical and Genetic Spectrum of Bartter Syndrome Type 3,” Journal of the American Society of Nephrology: JASN 28, no. 8 (2017): 2540–2552, 10.1681/ASN.2016101057. 28381550 PMC5533235

[17] L. F. Menegon , T. N. Amaral , and J. A. R. Gontijo , “Renal Sodium Handling Study in an Atypical Case of Bartter's Syndrome Associated With Mitochondriopathy and Sensorineural Blindness,” Renal Failure 26, no. 2 (2004): 195–197, 10.1081/JDI-120038522. 15287206

[18] R. Verma , A. Qavi , S. Pandey , and A. Bansod , “Bartter's Syndrome: A Rare Cause of Seizures and Quadriparesis,” Neurology India 65, no. 1 (2017): 184–185, 10.4103/0028-3886.198190. 28084271

[19] S. Patra , M. C. Konar , R. Basu , A. K. Khaowas , S. Dutta , and D. Sarkar , “Status Epilepticus as the Only Presentation of the Neonatal Bartter Syndrome,” Indian Journal of Endocrinology and Metabolism 16, no. 2 (2012): 300–302, 10.4103/2230-8210.93775. 22470874 PMC3313755

